# Antimicrobial resistance and microbiological gap analysis for central nervous system-assocaited bacterial pathogens in Nigeria

**DOI:** 10.3389/fmicb.2026.1695489

**Published:** 2026-06-16

**Authors:** Jafar Ammoura, Nouruldeen Alshamaa, Amjad Elamin, Ramadhani Chambuso

**Affiliations:** 1 Pathological Sciences Department, Division of Microbiology, College of Medicine, Ajman University, Ajman, United Arab Emirates; 2 Department of Global Health and Population, Harvard T.H. Chan School of Public Health, Harvard University, Boston, MA, United States

**Keywords:** African meningitis belt, antimicrobial resistance (AMR), bacterial pathogen, central nerous system, gap analysis, global health

## Abstract

**Background:**

Despite extensive vaccination efforts, central nervous system (CNS) infections remain a significant cause of morbidity and mortality in the African meningitis belt. This burden is increasingly complicated by the rising antimicrobial resistance (AMR) in pathogens not covered by the available vaccines. We mapped pathogen-specific AMR profiles and diagnostic gaps in CNS bacterial pathogens to inform precise microbiology-based interventions for improved surveillance and empiric therapy in Nigeria.

**Methods:**

We conducted a retrospective analysis of a three-year national AMR surveillance data, focusing on CNS bacterial pathogens. Data from 25 sentinel laboratories were extracted, analysed and interpreted per CLSI/GLASS standards. AMR profiles and diagnostic gaps were assessed using descriptive statistics and Chi-square tests for demographic and temporal comparisons.

**Results:**

Among 84,548 valid cultures from over 26,000 patients, culture positivity rate was higher in females (35.1%) compared to males (28.3%, *p* < 0.001) and higher among older adults (>65 years, 40.0%, p < 0.001). CSF specimens were underrepresented, while species-level ambiguity was high for CNS-associated infections. *Staphylococcus aureus* (31.3%), *Escherichia coli* (20%), and *Klebsiella species* (12%) were the dominant isolates, while *Pseudomonas aeruginosa* (3.5%) and *Acinetobacter species* (0.6%) showed persistent low-level presence. High levels of penicillin resistance were observed in *Streptococcus pneumoniae* across the study years, culminating at 100% in 2018. Other pathogens, including *Klebsiella species*, *E. coli*, and *P. aeruginosa,* showed high AMR across multiple drug classes. Gap analysis scored all CNS-associated bacterial pathogens at maximum clinical risk (5/5), with major deficits in detection and the laboratory capacity. A precision-targeted recommendation map proposed tailored microbiological interventions, such as neonatal antimicrobial susceptibility testing (AST) protocols for *E. coli* and intensive care unit infection registries for *Pseudomonas* species.

**Conclusion:**

Species-level identification gaps and high AMR in CNS bacterial pathogens demand targeted microbiological-led diagnostics, including expanded cerebrospinal fluid testing and AST-guided empiric therapy in resource-limited settings.

## Graphical abstract

**Figure:**
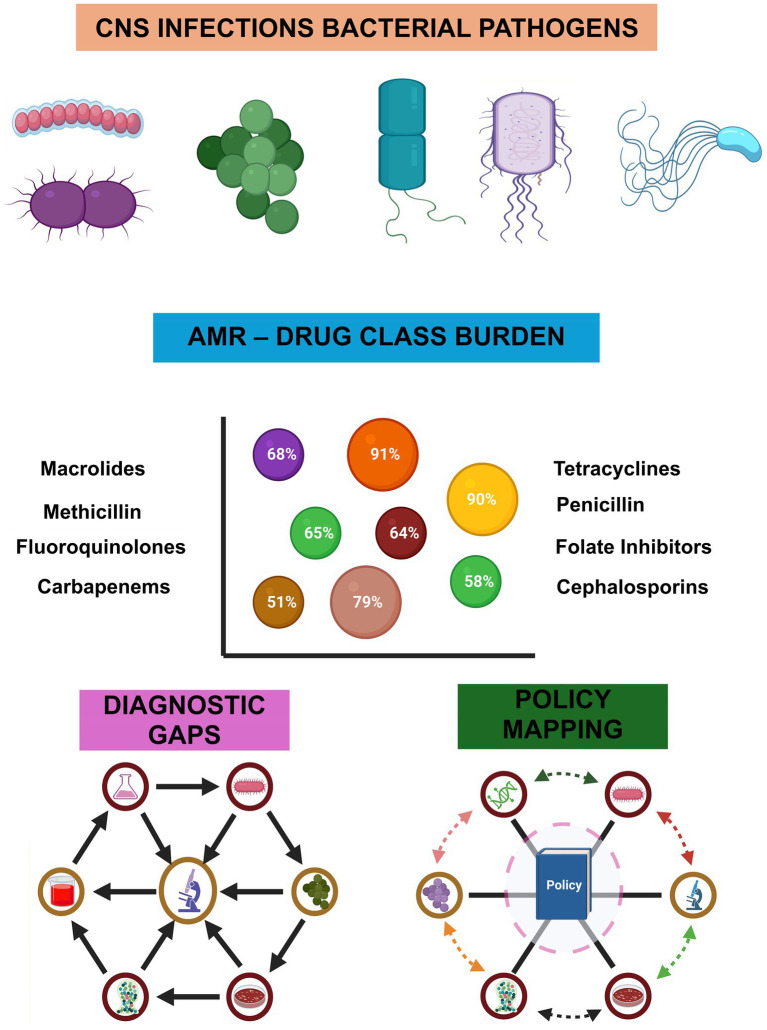

## Highlights

- First national AMR gap analysis for CNS pathogens across 84,000 cultures in Nigeria
- Links resistance trends with diagnostic gaps for tailored stewardship interventions
- Introduces scalable microbiology-driven precision surveillance for LMIC settings
- Informs treatment protocols, lab policies and strategies for high-risk CNS infections

## Introduction

Central nervous system (CNS) infections are a group of serious and potentially life-threatening conditions that affect the brain, spinal cord, optic nerves, and their covering membranes (Giovane and Lavender, 2018). The bacterial CNS infections cause over 318,000 deaths globally each year, with sub-Saharan Africa accounting for more than half of this burden concentrated in the “African Meningitis Belt” which includes countries such as Nigeria, the most populous country within the “African Meningitis Belt” (Barichello et al., 2023; Reese et al., 2019) For instance, between October 2022 and April 2023, Nigeria reported 1,686 suspected meningitis cases with 124 deaths, a case fatality rate of 7% (WHO, 2023). Existing vaccines such as the MenAfriVac have substantially reduced serogroup A *meningococcal* disease (Sherman and Stephens, 2020; Trotter et al., 2017). However, other CNS bacterial infections remain driven by non-vaccine serogroups and resistant pathogens like *Streptococcus pneumoniae*, *Escherichia coli*, and *Klebsiella pneumoniae*, where empirical treatment failure is increasingly linked to antimicrobial resistance (AMR) (AMR-Collaborators, 2024; Le Govic et al., 2022; WHO, n.d.; WHO, 2021).

AMR is a parallel emergency in CNS bacterial infections, reducing the effectiveness of penicillins, cephalosporins, and fluoroquinolones, drugs essential for empirical therapy (Ondoa et al., 2025). Empirical therapy is further compromised by the absence of detailed national AMR microbiological mapping for CNS bacterial pathogens (Uddin et al., 2021; Guo et al., 2022). Bacterial pathogens such as *Acinetobacter baumannii* and *Pseudomonas aeruginosa* are less frequently reported in community-acquired CNS infections but are well recognized in healthcare-associated and device-related CNS infections (Elfadadny et al., 2024; Mączyńska et al., n.d.). There are also gaps in species-level identification and laboratory capacity, leaving surveillance for CNS bacterial infections fragmented. Fragmentation has prevented a systematic gap analysis of CNS bacterial pathogens and AMR profiles (Moore et al., 2024; Chukwumeze et al., 2021). This resulted in a novel question on what are the pathogen-specific diagnostic, AMR, and existing microbiological gaps undermining effective management of CNS bacterial infections, to address a critical and long-standing knowledge void (Chaelin et al., 2023; Robertson et al., 2019).

The identified knowledge void limits understanding of diagnostic blind spots and AMR profiles across high-priority CNS-associated bacterial pathogens (Jesudason, 2024). Without this knowledge, targeted interventions and empiric therapy will remain unfeasible and misaligned (Kraef et al., 2024). Also, species-level detection is particularly important to address any rising resistance among neuroinvasive pathogens, which further complicates the management of CNS infections (Brouwer and van de Beek, 2017). We hypothesize that bacterial pathogens associated with CNS infections are disproportionately affected by detection capacity and improper AMR profiles which can be systematically mapped to inform microbiology-driven precision AMR strategies, tailored for national infection control programs. To address our hypothesis from a global health perspective, we performed a microbiology-driven gap-analysis study of AMR in CNS infections for associated bacterial pathogens across a large-scale national surveillance data from Nigeria.

## Methodology

### Ethics statement

This study utilized publicly available, de-identified AMR surveillance data originally collected by the Nigerian Ministry of Health under the Fleming Fund Regional Grant (Phase 1) https://aslm.org/wp-content/uploads/2023/07/AMR_REPORT_NIGERIA.pdf?x89467. No individual-level data were accessed. Secondary data use complied with the Declaration of Helsinki and did not require additional ethical clearance.

### Study design

We conducted a retrospective study of the national AMR surveillance report of Nigeria for the years 2016 to 2018. This report, published in 2022, was generated through the Mapping Antimicrobial Resistance and Antimicrobial Use Partnership (MAAP) and involved 25 sentinel laboratories across Nigeria with bacteriology testing capacity. This study adheres to the STROBE (Strengthening the Reporting of Observational Studies in Epidemiology) guidelines.

### Data sources and surveillance framework

The original data were collected by Nigerian health authorities in collaboration with MAAP partners. Surveillance activities were conducted across a national network of medical laboratories selected for their bacteriology testing capacity. A total of 25 laboratories contributed antimicrobial susceptibility testing (AST) data using WHONET software. Trained field teams retrieved laboratory records from both paper-based and digital systems. Where feasible, these records were linked with hospital databases for clinical metadata, including age, sex, and specimen type. Specimens for culture were collected from cerebrospinal fluid (CSF), blood, urine, and other clinical sources (e.g., abscess, discharge, wound swabs). The present analysis focused on CNS-associated bacterial pathogens across all specimen types within the national surveillance dataset. This is because specific pathogens with neuroinvasive potential may originate at distant sites and subsequently seed the CNS and cause infections via hematogenous spread.

### Data extraction

From the national AMR report, the following data were extracted:

- Total number of participated laboratories
- Collected specimens per year
- Source of collected specimens
- Specimen type
- Valid cultures
- Positive and negative cultures
- Species-level breakdown
- AST results for antibiotics tested
- Patient demographics (age and sex)

These variables were compiled into a structured dataset to allow for year-by-year resistance analysis. The complete extracted variable list used for this analysis is provided in Supplementary Table 1.

### CNS infections bacterial pathogens

While the surveillance report captured a broad range of pathogens, this study specifically focused on the most prevalent and clinically significant CNS infections-associated pathogens observed in the report. These organisms cause bacterial meningitis, neonatal sepsis, brain abscesses, ventriculitis, and other CNS-related conditions in both adult and paediatric populations (WHO, 2021; van Soest et al., 2023; Pai et al., 2015; Rodríguez-Lucas et al., 2020; Sun et al., 2021; Moussiegt et al., 2022; Sayyahfar et al., 2021). This included:

a Common CNS infections bacterial pathogens

- *Neisseria meningitidis.*
- *Haemophilus influenzae.*
- *Streptococcus species*: *Streptococcus pneumoniae and Streptococcus.*
- *agalactiae.*

Rare CNS infections bacterial pathogens

- Staphylococcus species: *Staphylococcus aureus* and *Staphylococcus epidermidis*
- Pseudomonas species: *Pseudomonas aeruginosa*.
- Klebsiella species: *Klebsiella pneumoniae*, *Klebsiella oxytoca* and Klebsiella aerogenes.
- Escherichia species: *Escherichia coli*
- Acinetobacter species: *Acinetobacter baumannii*, *Acinetobacter haemolyticus* and *Acinetobacter lwoffii*
- Other Streptococcus species such as: *Streptococcus pyogenes*, Streptococcus viridans, *Streptococcus anginosus*, *Streptococcus bovis*, Streptococcus gallolyticus, *Streptococcus gordonii*, Streptococcus milleri, *Streptococcus mitis*, *Streptococcus oralis*, *Streptococcus parasanguinis*, *Streptococcus salivarius*, *Streptococcus sanguinis* and *Streptococcus suis*.

### Inclusion criteria and data management

Only isolates from the above list with valid AST results were included in the analysis. In accordance with WHO Global AMR Surveillance System (GLASS) guidelines and Clinical and Laboratory Standards Institute (CLSI) M39-A4 recommendations, only the first isolate per patient per year was retained to prevent duplicate entries (WHO, 2022; Pierce and Mathers, 2022). When unique patient identifiers were unavailable, all isolates were retained and interpreted with caution. AST interpretations followed CLSI guidelines, and WHONET interpretive rules were applied to ensure consistent reporting across laboratories (Dikkatwar and Vaghasiya, 2023).

### AST and quality control

AST was performed using disk diffusion and MIC panels, depending on laboratory capacity, as described in the original report (https://aslm.org/wp-content/uploads/2023/07/AMR_REPORT_NIGERIA.pdf?x89467). CLSI standards were applied across all laboratories. Internal quality control was conducted using standard QC strains. External quality assessment (EQA) was implemented through structured proficiency testing coordinated by MAAP and the Fleming Fund. Although laboratories were encouraged to participate regularly, specific participation rates and EQA performance outcomes were not consistently detailed in the original report.

### AMR calculation

From the original report, AMR rates were derived from positive cultures with available AST results. AMR rates were calculated as the proportion of non-susceptible isolates (intermediate or resistant) relative to the total number of tested isolates within a single calendar year.


$$\begin{array}{c} \mathrm{AMR}\;\text{Rate}\;(\%)=(\frac{\text{Number of non}-\text{susceptible}\;\;\text{isolates}\;}{\text{Number of isolates tested for antimicrobial susceptibility}})\times 100\\ \left(95\%\text{Confidence interval}\right) \end{array}$$

Where:

- *Non-susceptible isolates* = Resistant + Intermediate
- *Tested isolates* = All isolates subjected to AST

### Statistical analysis

Analyses were carried out in R version 4.3.2. Because less than 1% of the data was missing, a complete-case analysis was deemed sufficient. Valid culture data were analyzed using descriptive statistics to explore positivity rates by demographic group and by year and Wald confidence intervals (CI) were calculated. Pearson’s Chi-square test was used for categorical comparisons, with *p* < 0.05 considered statistically significant.

## Results

### Demographics and culture characteristics

Among the 84,548 valid cultures processed across all laboratories for all specimens, culture positivity was notably higher in females (35.1%) than males (28.3%), with a significant statistical difference across gender groups (*p* < 0.001). Older age groups showed increased positivity, rising from 23.9% in children aged 1–17 years to 40.0% in those above 65 years, with the highest yield seen in the unknown age group (42.5%; *p* < 0.001). Over the years, culture positivity steadily increased from 28.5% in 2016 to 34.6% in 2018 (*p* < 0.001), suggesting improved diagnostic recovery or changing epidemiology (Table 1).

**Table 1:** Diagnostic yield across all cultures.

Variable	Group	Valid cultures, *n*	Positive cultures with AST, *n*	Negative cultures, *n* (%)	Positive cultures, *n* (%)	Negative cultures, *n* (%)	95% CI for Positive (%)	*p-value
Gender	Male	37,550	10,160	26,932 (71.7%)	10,618 (28.3%)	26,932 (71.7%)	(27.8–28.7)	**<0.001**
	Female	46,997	13,803	30,480 (64.9%)	16,517 (35.1%)	30,480 (64.9%)	(34.7–35.6)	
Age	< 1 year	9,689	2,711	6,857 (70.8%)	2,832 (29.2%)	6,857 (70.8%)	(28.3–30.1)	**<0.001**
	1–17 yrs	20,476	4,526	15,714 (76.7%)	4,902 (23.9%)	15,714 (76.7%)	(23.4–24.5)	
	18–49 yrs	29,336	7,629	20,013 (68.2%)	9,323 (31.8%)	20,013 (68.2%)	(31.2–32.3)	
	50–65 yrs	5,177	1,630	3,424 (66.1%)	1753 (33.9%)	3,424 (66.1%)	(32.6–35.2)	
	> 65 yrs	4,743	1806	2,845 (60.0%)	1898 (40.0%)	2,845 (60.0%)	(38.6–41.4)	
	Unknown	15,127	5,661	8,700 (57.5%)	6,427 (42.5%)	8,700 (57.5%)	(41.7–43.3)	
Year	2016	26,896	6,532	19,218 (71.5%)	7,678 (28.5%)	19,218 (71.5%)	(28.0–29.1)	**<0.001**
	2017	32,134	9,501	21,494 (66.9%)	10,640 (33.1%)	21,494 (66.9%)	(32.6–33.6)	
	2018	25,518	7,930	16,701 (65.4%)	8,817 (34.6%)	16,701 (65.4%)	(34.0–35.1)	

*Pearson’s χ^2^ test comparing the distribution of positive versus negative cultures across all categories within each variable. Significant *p*-values are bolded.

### Diagnostic yield, specimen distribution and temporal trends of all cultures across participating laboratories

**Figure 1:**
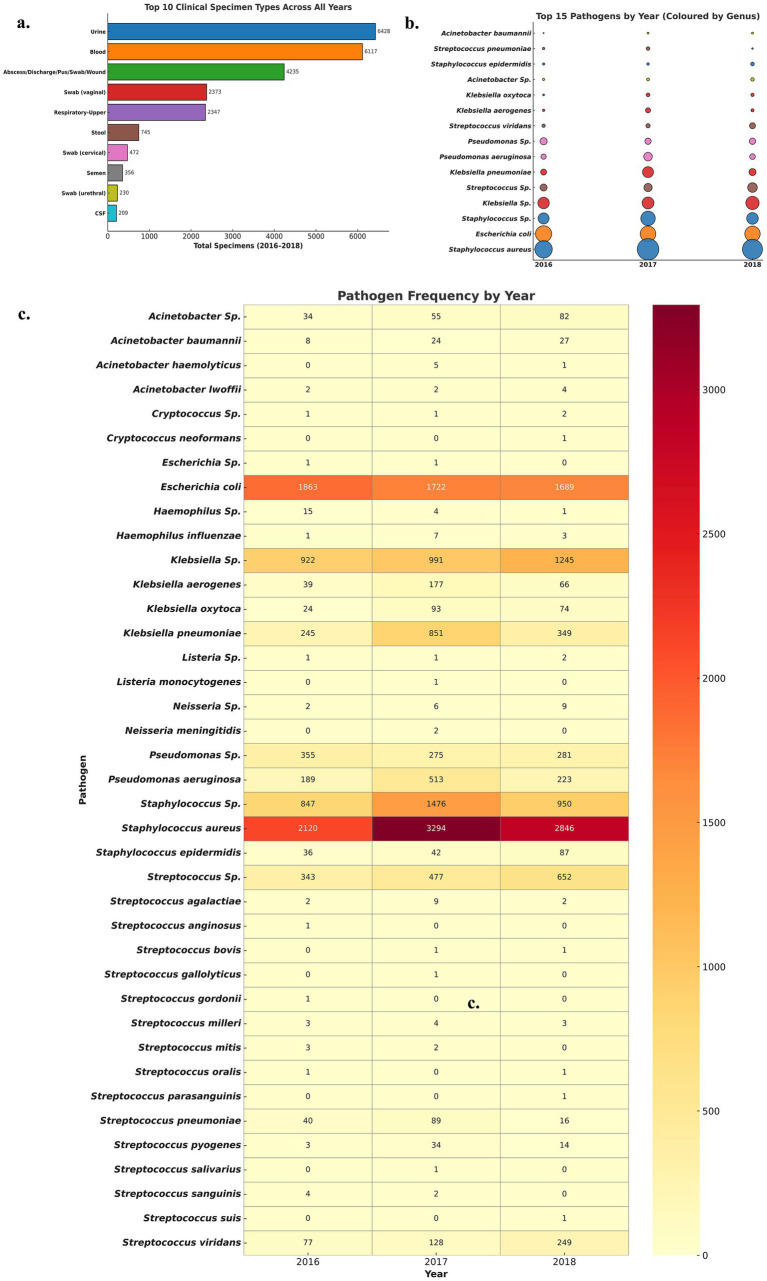
Diagnostic and epidemiological landscape across laboratories and specimen types in Nigeria. **(a)** Aggregate specimen mix across all years, with urine and blood predominating; abscess/pus/wound samples also contributed substantially, whereas CSF submissions are rare, limiting inference for CNS infections. **(b)** Heatmap of annual pathogen frequencies, identifying *Staphylococcus aureus* and *Escherichia coli* as dominant across years, with year-to-year fluctuations in *Klebsiella pneumoniae* and *Pseudomonas aeruginosa*. **(c)** Top 15 pathogens by year (colored by genus), demonstrating consistent predominance of *S. aureus*, *E. coli*, and *Klebsiella* species.

There were critical diagnostic gaps in CNS specimen collection and species resolution, particularly for high-priority neurological pathogens (Figure 1; Supplementary Figure 1). We observed substantial inter-laboratory variability in diagnostic and AST coverage, with some high-volume centers exceeding 90% AST reporting while others, such as Bwari and FNHI Yaba, showed critical shortfalls (Supplementary Figure 1a). Among specimen types, CSF accounted for only 209 samples, making it the least frequently submitted despite its importance for CNS diagnostics (Figure 1a; Supplementary Figure 1b). *Cryptococcus neoformans*, *N. meningitidis,* and *Listeria monocytogenes*, all key CNS pathogens were rarely isolated (Figure 1b). Species-level ambiguity was high for *Neisseria* (95%) and *Cryptococcus* (90%), severely limiting diagnostic confidence in CNS infections (Supplementary Figure 1e). *S. aureus* remained the leading isolate overall, peaking in 2017, while *E. coli* and *K. pneumoniae* showed consistent but variable prevalence (Figures 1b,c; Supplementary Figure 1d). Although *P. aeruginosa* and *A. baumannii* were less frequent, their stable presence signals persistent risk of nosocomial CNS infections (Ogbolu et al., 2020).

### Yearly AMR patterns across drug classes and specific CNS-associated bacterial species

**Figure 2:**
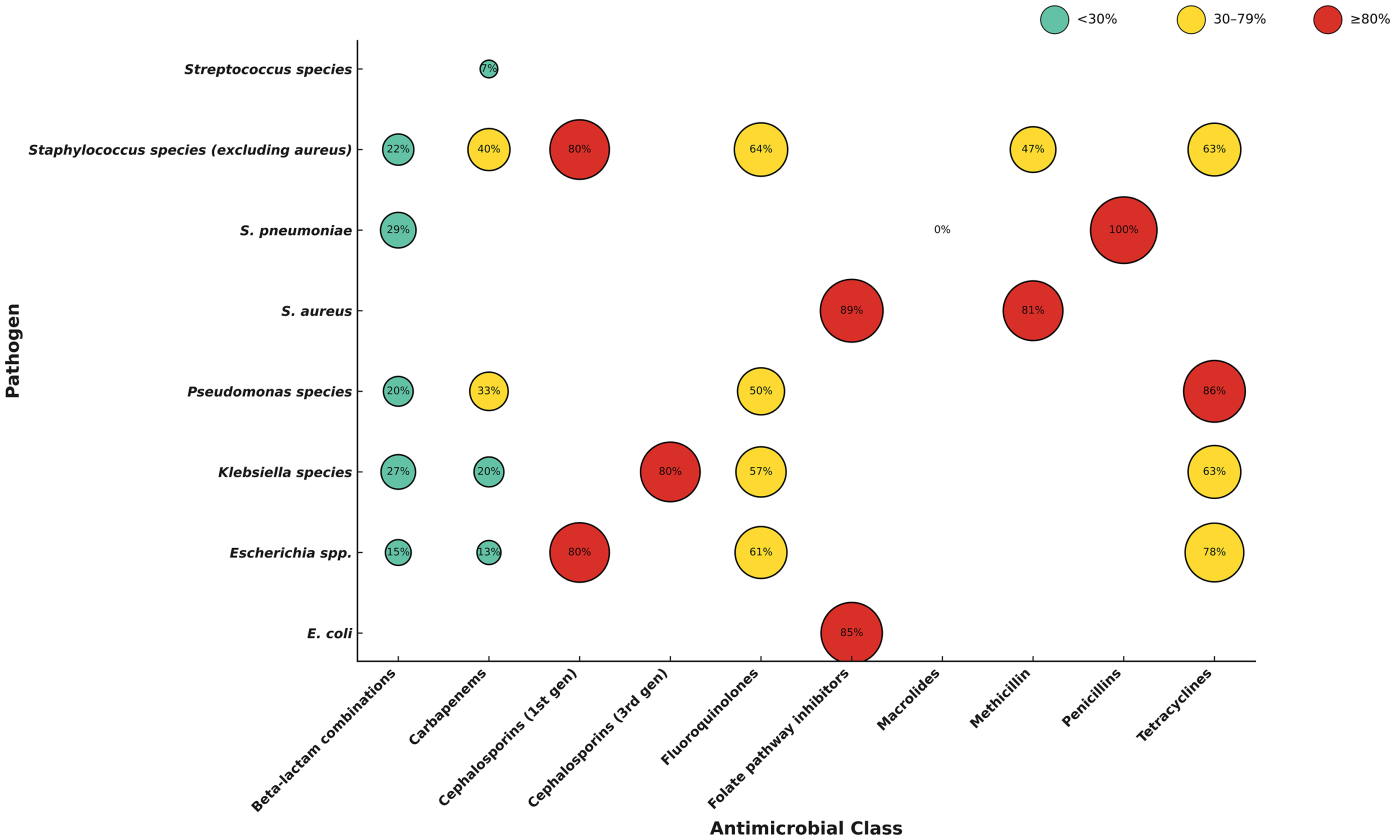
AMR profiles of CNS-associated pathogens across different drug classes. Bubble plot for 2018 showing pathogen-specific resistance across antimicrobial classes, including complete (100%) penicillin resistance in *Streptococcus pneumoniae* and high resistance among other CNS-associated pathogens, signaling critical gaps in effective empirical therapy for CNS and systemic infections.

The AMR among key CNS-associated bacterial pathogens revealed escalating threats to empirical treatment, particularly for *S. pneumoniae*, a major cause of meningitis, it exhibited alarmingly high to complete resistance to penicillin, rising from 67% in 2016 to 100% in 2018, alongside persistently elevated macrolide resistance (>70%), undermining first-line CNS therapy (Figure 2; Supplementary Figure 2). *S. aureus* and *E. coli* showed high resistance to folate pathway inhibitors. Among Gram-negative pathogens, *Klebsiella* species displayed high resistance to 3rd generation cephalosporins. *Pseudomonas* species showed high resistance to tetracyclines (86%).

### AMR analysis for CNS pathogens to address critical microbiological gaps

**Figure 3:**
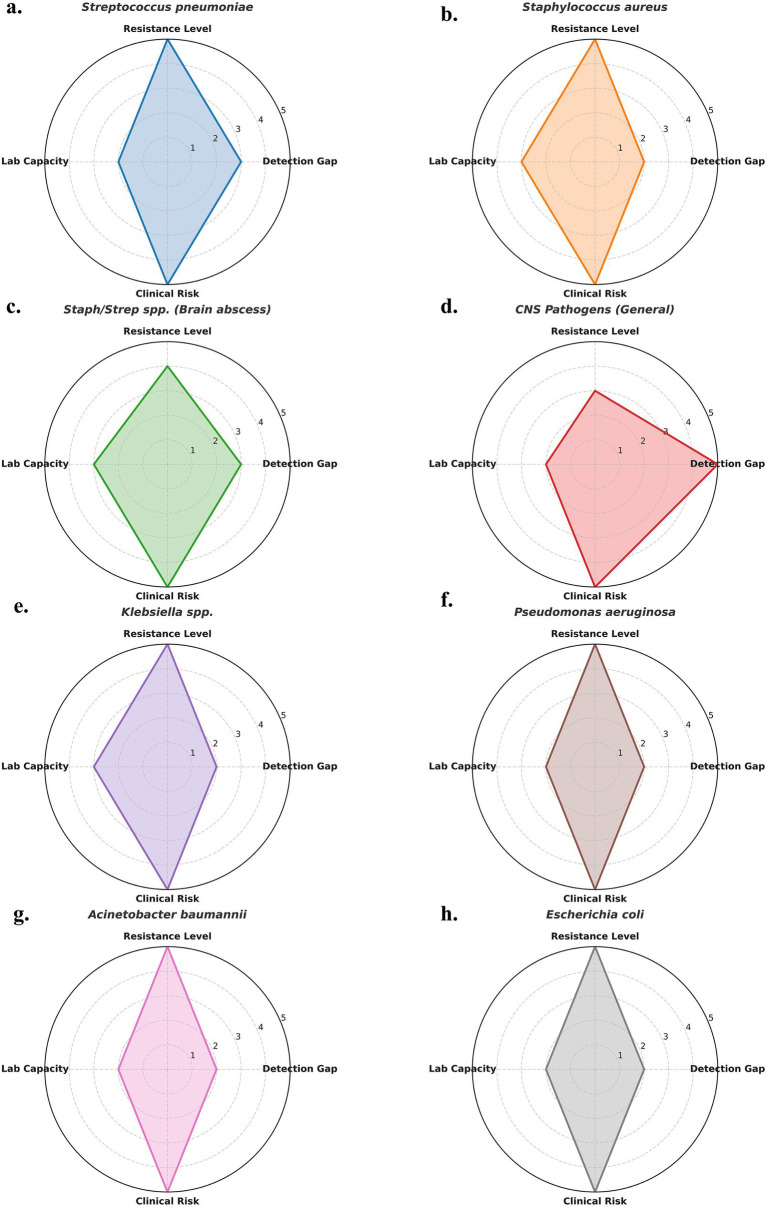
Pathogen-specific gap analysis for CNS-associated high-risk organisms across four domains: detection gap, resistance level, laboratory capacity, and clinical risk. **(a)** *Streptococcus pneumoniae* shows maximal resistance and clinical risk scores (5/5), with poor laboratory capacity (2/5) and a moderate detection gap (3/5). **(b)** *Staphylococcus aureus* shows moderate lab capacity in screening and low detection gap. **(c)** *Staph/Strep* spp. associated with brain abscesses exhibited high clinical risk and resistance level but are affected by moderate detection and lab capacity. **(d)** CNS pathogen surveillance scores worst in detection (5/5) and lab capacity (2/5). **(e)** *Klebsiella* species demonstrate high resistance (5/5) and clinical risk and low detection gap. **(f)** Pseudomonas aeruginosa exhibits uniformly high resistance and clinical risk with low detection and lab gaps (2/5 each). **(g)** *Acinetobacter baumannii* shows equally elevated resistance and clinical risk scores with low detection and lab-capacity gaps. **(h)** *Escherichia coli dis*plays high resistance levels and clinical risk while showing lab and detection limitations.

Gap analysis across eight CNS-associated pathogens revealed critical deficiencies in detection, resistance profiling, and laboratory capacity, with consistently high clinical risk scores (5/5) across all organisms (Figure 3). *S. pneumoniae* and *S. aureus* scored maximally for resistance and risk, reflecting the burden of penicillin-resistant meningitis and invasive MRSA infections, respectively, both compounded by low lab capacity. CNS device-associated pathogens (*Staph/Strep* species) and *A. baumannii* also demonstrated high resistance and inadequate detection infrastructure, highlighting vulnerabilities in neurosurgical infection control in Nigeria (Ogbolu et al., 2020). Surveillance for CNS pathogens overall scored worst in detection gap (5/5), exposing systemic underreporting and incomplete data across sentinel laboratories. *Klebsiella* species and *E. coli*, both linked to severe neonatal CNS infections, showed extreme resistance (5/5). Both *P. aeruginosa* and *Acinetobacter* species showed elevated resistance and CNS risk, further reinforcing the urgent need for targeted diagnostics and empiric therapy realignment in CNS infections.

### Addressing pathogen-specific challenges in CNS infections

**Figure 4:**
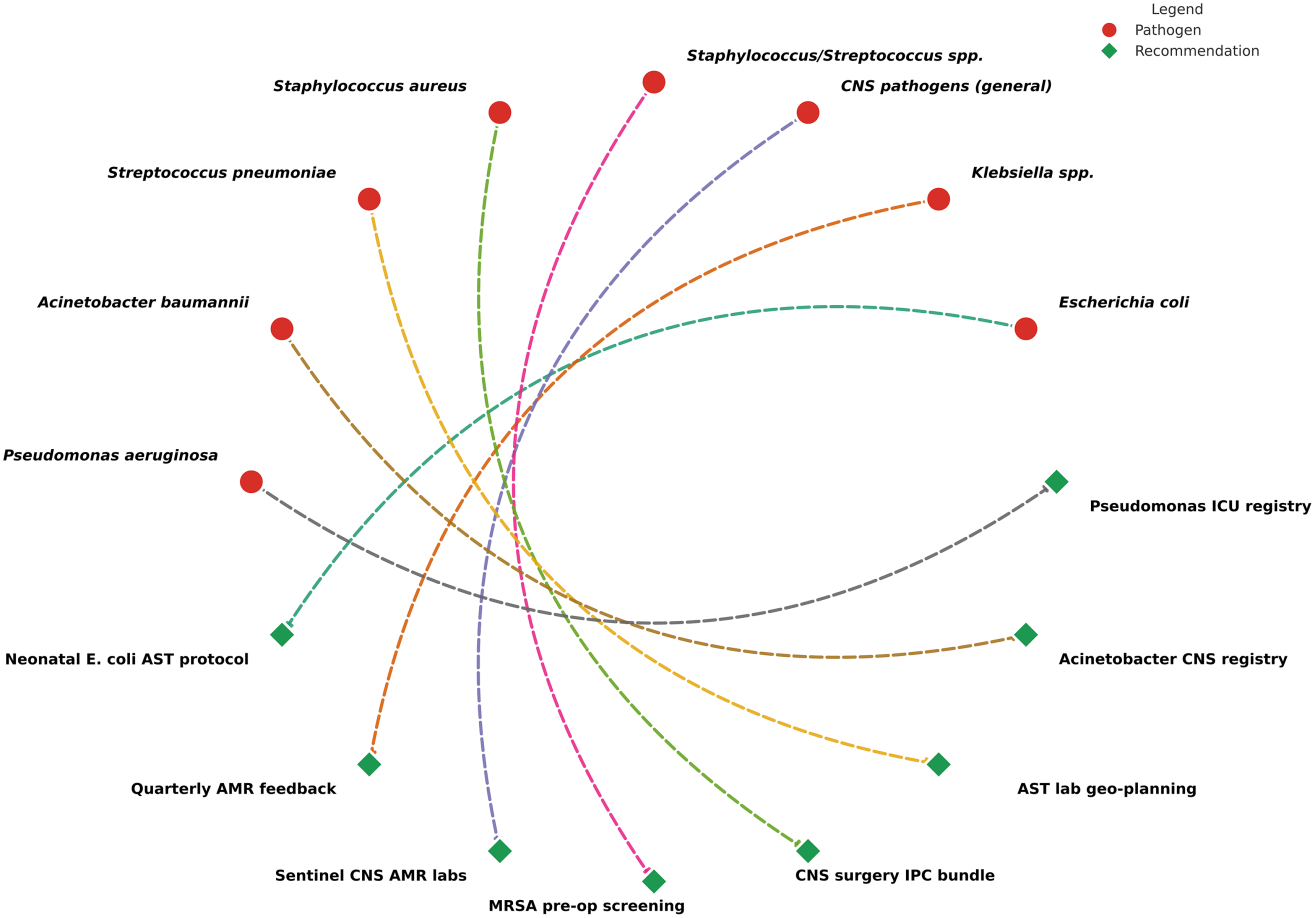
Pathogen-specific recommendations for AMR strategies in CNS infections. A circular network maps eight CNS-associated pathogens to tailored microbiological interventions. Recommendations include neonatal AST protocols for *Escherichia coli*, a CNS registry for *Acinetobacter baumannii*, and ICU-based CNS infection registries for *Pseudomonas aeruginosa*. Cross-cutting gaps were addressed by linking general CNS pathogens to sentinel AMR laboratories with molecular diagnostics.

We explored a precision surveillance strategy, visually aligning pathogen biology with targeted diagnostics, stewardship, and infection prevention across diverse CNS clinical settings (Figure 4). This was performed to provide a strategic, organism-specific action model to inform diagnostic stewardship and infection prevention frameworks for CNS infections in high-burden, resource-limited settings. Targeted intervention mapping across eight high-priority CNS-associated pathogens revealed key microbiology-informed gaps in AMR response systems. *E. coli*, frequently implicated in neonatal meningitis, was linked to neonatal AST protocol initiation, while *Klebsiella* species and *P. aeruginosa*, both frequently implicated in post-surgical CNS infections, quarterly AMR feedback and ICU-based infection registries were recommended, respectively. For *A. baumannii*, a persistent MDR pathogen in neurocritical care, initiation of a CNS registry was recommended (Ogbolu et al., 2020). Gram-positive cocci including *S. aureus* and *S. pneumoniae* were distinctly tied to IPC bundles and AST lab geo-planning, reflecting their dual role as colonizers and invasive agents. Notably, CNS-wide diagnostic gaps were addressed through the inclusion of sentinel AMR laboratories, emphasizing a systems-level response to data fragmentation.

## Discussion

This study provides the first integrated microbiological-driven gap-analysis assessment of AMR in CNS infection-associated pathogens across a large-scale national surveillance platform in Nigeria, encompassing over 84,000 cultures. By linking organism-specific resistance trends, diagnostic performance gaps, and pathogen-tailored recommendations, our study introduces a precision AMR surveillance model uniquely adapted for resource-limited settings. The novel use of structured gap analysis and creative intervention mapping for CNS infection-associated pathogens including *E. coli*, *Klebsiella* species, *P. aeruginosa*, *S. aureus* and *S. pneumoniae* translates complex laboratory surveillance data into actionable practical tools such as neonatal AST protocols, ICU infection registries, and IPC bundles. These findings offer a scalable framework to inform empiric CNS infections treatment guidelines, optimize lab resource allocation, and strengthen national AMR policy. Given the global urgency of rising AMR in CNS pathogens and the absence of structured diagnostic and stewardship strategies in many LMICs, this study fills a critical evidence gap and has direct implications for regional and global preparedness for CNS infections.

The current study emphasizes on the profound microbiological vulnerabilities in the surveillance and management of CNS infections, particularly in how diagnostic insufficiencies amplify the impact of AMR in Nigeria (Ogbolu et al., 2020; Ajekiigbe et al., 2025). The dominance of non-speciated pathogen genera reflects not only a failure of species-level diagnostics but also a missed opportunity to implement pathogen-specific treatment and public health interventions, especially for non-vaccine-preventable infections. High and rising resistance rates in *S. pneumoniae* and *Klebsiella* species suggest selective pressure from empiric therapy regimens that may be poorly matched to local susceptibility profiles, highlighting the urgency for local antibiogram-driven prescribing (Lestin-Bernstein et al., 2021). In addition, organisms traditionally considered secondary in CNS infections, such as *A. baumannii* and *P. aeruginosa*, maintained a stable presence in the dataset. These pathogens are predominantly linked to healthcare-acquired infections, particularly in intensive care and device-related contexts, and their persistence in this surveillance framework may reflect underlying nosocomial infection dynamics (Ogbolu et al., 2020; Karaaslan et al., 2025). Moreover, the high prevalence of resistance to critical antimicrobial drug classes such as cephalosporins, tetracyclines, and folate inhibitors across pathogens associated with CNS infections reflects the existing challenges facing antimicrobial armamentarium in both Gram-positive and Gram-negative CNS- associated pathogens (Chinemerem Nwobodo et al., 2022; Mancuso et al., 2021). In our study, we have exposed this significant microbiological landscape characterized by incomplete species resolution, over-reliance on empiric broad-spectrum therapy, and inadequate alignment between laboratory evidence and clinical decision-making, challenges that are central to both treatment failure and the acceleration of resistance globally.

The results of this study align with and extend prior regional AMR surveillance efforts while revealing critical divergences that expose the limitations of previously available data. For instance, the WHO GLASS report (2022) acknowledges rising resistance in *S. pneumoniae* and *K. pneumoniae* globally, but our data show that *S. pneumoniae* resistance to penicillin in CNS infections reached 100% by 2018, significantly higher than the 45–60% reported in aggregated GLASS datasets for sub-Saharan Africa (WHO, 2022). This discrepancy likely reflects the inclusion of rare CNS-associated isolates in global estimates and highlights the unique resistance pressure within CNS compartments, where blood–brain barrier pharmacokinetics and delayed diagnosis may drive more aggressive antibiotic use (Haddad et al., 2022; Banks et al., 2024; Alajangi et al., 2022). Similarly, our finding of 86% tetracycline resistance in 2018 for *Pseudomonas* species is considerably higher than the 53% resistance rate previously reported in Nigeria, likely reflecting nosocomial CNS-specific strains associated with device colonization and prolonged ICU stays (Ogbolu et al., 2020; Ogbolu et al., 2020; Ugbo et al., 2025; Irekeola et al., 2025). The diagnostic gaps observed here, especially the 95% species-level ambiguity contrasts sharply with surveillance systems in high-income countries such as CDC’s Active Bacterial Core Surveillance (ABCs), which report >95% speciation accuracy due to universal adoption of PCR-based diagnostics and centralized data harmonization. Moreover, while most African AMR studies focus on urinary or bloodstream isolates, our CNS-specific gap analysis study uniquely exposes the underrepresentation of CSF samples and the lack of tailored response strategies for neurological infections. These differences emphasize the contextual nature of AMR dynamics and the need for surveillance systems that capture niche infection types and high-risk anatomical sites. The convergence of high resistance, diagnostic fragmentation, and clinical risk in our findings thus presents a distinct epidemiological profile that current regional and global frameworks fail to adequately capture (Alabi et al., 2025).

The pathogen-specific gap analysis conducted in this study revealed not only the extent of diagnostic and resistance challenges but also offered a structured framework for microbiologically informed intervention. By quantifying detection gaps, laboratory capacity limitations, and clinical risk across eight CNS-associated pathogens, the analysis exposed consistent deficits that disproportionately affect organisms with high virulence and poor therapeutic coverage. For instance, *S. pneumoniae* and *S. aureus* exhibited maximal resistance and risk scores, yet were matched with suboptimal laboratory capacity, pointing to an urgent need for targeted AST strengthening and IPC programs. The translational strength of this study lies in its alignment of these quantitative gaps with actionable microbiological solutions, such as neonatal-specific *E. coli* AST protocols, *Acinetobacter-*specific CNS surveillance registries, and geospatial AST lab expansion for high-burden regions (Ogbolu et al., 2020). Unlike generic AMR strategies, these recommendations are pathogen-matched and informed by real surveillance data, offering governments and hospital networks a prioritization model to guide resource allocation and diagnostic scale-up (Coque et al., 2023; Ahmed et al., 2024). Our approach transforms abstract surveillance outputs into a practical toolkit for strengthening empiric therapy frameworks, improving early detection of high-risk CNS infections, and institutionalizing precision AMR responses in neurosurgical and neonatal care settings.

The strengths and limitations that distinguish this study include:

This study represents the first large-scale CNS-focused AMR surveillance analysis using secondary national data incorporating more than 84,000 culture results with pathogen-specific AMR across multiple years and laboratories. The integration of laboratory analysis of diagnostic yield, species resolution, and AST coverage exposes systemic weaknesses often missed in pathogen-only studies. The use of a structured, pathogen-specific gap analysis quantifies detection, resistance, laboratory capacity, and clinical risk across eight CNS-associated pathogens in Nigeria, introducing a novel scoring framework for prioritizing interventions. The introduction of precision intervention mapping aligns CNS-associated pathogens with tailored, microbiology-informed recommendations. Our CNS infection-specific AMR patterns linked to empirical treatment implications, demonstrating high-level resistance in *S. pneumoniae*, *Klebsiella* species, and *Pseudomonas* species, directly challenge current first-line CNS infection therapies in LMICs. The focus on underrepresented CNS-associated pathogens from high-risk clinical samples (CSF) highlights the diagnostic neglect of CSF and CNS infections in national surveillance systems, guiding reconsideration of clinical sample collection and laboratory policy. Furthermore, the study provides data-driven tools to guide policymakers in laboratory investment, empiric guideline development, and targeted infection control interventions for CNS infections, and offers a scalable framework adaptable to other LMICs seeking to build CNS pathogen surveillance within fragmented or underfunded clinical and laboratory networks.

However, several limitations should be acknowledged. There was underrepresentation of CSF specimens, as CNS infections were poorly captured due to the extremely low volume of CSF submissions, limiting pathogen diversity and clinical correlation. High species-level ambiguity for multiple CNS pathogens undermined diagnostic specificity and epidemiological validity. Inter-laboratory heterogeneity in AST coverage, marked by differences in reporting rates across laboratories, may have introduced potential biases in resistance estimates and reduced comparability. The absence of clinical outcome data (e.g., mortality, treatment response) restricts the ability to evaluate the direct impact of AMR patterns on patient care and treatment outcomes. The cross-sectional retrospective design and use of aggregated yearly data limit the ability to assess temporal causality or emergence of specific resistance clones over time. Limited molecular diagnostic data and absence of genotypic or PCR-based confirmation preclude detection of resistance genes, virulence factors, or outbreak clusters. Additionally, the surveillance dataset lacked detailed clinical metadata (e.g., timing of infection onset, hospitalization status, ICU exposure), precluding differentiation between community-acquired and hospital-acquired infections. Furthermore, the analysis was organism-focused rather than restricted to confirmed CNS specimen sources, which may limit direct attribution of resistance patterns specifically to CNS infection episodes.

Despite these limitations, this study offers critical and actionable insights by leveraging one of the largest CNS-focused culture datasets in the region, revealing resistance patterns and diagnostic gaps that have not been previously characterized. The use of robust microbiological analysis, cross-laboratory comparison, and pathogen-specific intervention mapping ensures that the findings remain valid and highly relevant for national AMR response planning. Moreover, the identified gaps themselves emphasize the urgent need for surveillance reform, making the results not only valuable but essential for guiding future diagnostic investments and empirical treatment policies in low-resource settings.

## Conclusion

This study provides the first national microbiology-centred secondary data analysis of surveillance and AMR among CNS infection-associated pathogens in Nigeria. It builds upon the foundational progress made by Nigerian public health authorities and surveillance laboratories by providing the first focused analysis of CNS infections through a microbiological and global health systems lens. It suggests that, despite substantial national investment in AMR surveillance in Nigeria, critical gaps persist in the detection, species-level identification, and antimicrobial susceptibility testing of high-priority CNS-associated pathogens. The findings highlight the urgent need to complement ongoing efforts with pathogen-specific, site-adapted strategies that strengthen laboratory capacity and empiric treatment guidance. By introducing a precision gap analysis and intervention model, our study offers a translational framework to enhance existing surveillance programs and inform national policy, without undermining the valuable progress already achieved. The key takeaway message is that national AMR response strategies must move beyond generic stewardship to pathogen-targeted, site-specific interventions. Immediate priorities should include expanding CSF diagnostics, adopting routine species-level identification and deploying AST-guided empiric therapy models, particularly for high-risk neurological and neonatal infections. These findings are scalable and urgently relevant to similar high-burden, resource-constrained settings across sub-Saharan Africa.

## Data Availability

The original contributions presented in the study are included in the article/Supplementary material, further inquiries can be directed to the corresponding author.
