# Supplementary material for: Antimicrobial resistance and microbiological gap analysis for central nervous system-assocaited bacterial pathogens in Nigeria
Source: Front Microbiol. 2026 Jun 16;17:1695489. doi: 10.3389/fmicb.2026.1695489 (PMC13314636; doi:10.3389/fmicb.2026.1695489)
Supplement: Supplementary file 1 [file Data_Sheet_1.DOCX]

**Supplementary Fig. 2. Supporting AMR profiles of CNS-associated pathogens across antimicrobial drug classes.** (a) Heatmap for 2016 showing AMR percentages by pathogen and antimicrobial class. (b) Bubble plot for 2016 showing widespread high resistance to third-generation cephalosporins and methicillin, with high fluoroquinolone resistance in Pseudomonas species. (c) Heatmap for 2017 showing AMR percentages by pathogen and antimicrobial class, including high resistance in Klebsiella aerogenes to second-generation cephalosporins. (d) Bubble plot for 2017 highlighting multidrug resistance patterns, including resistance in K. aerogenes, E. coli, and S. pneumoniae. (e) Heatmap for 2018 illustrating AMR percentages by pathogen and antimicrobial class, supporting the key resistance patterns shown in Fig. 2.
