# Supplementary material for: Antimicrobial resistance and microbiological gap analysis for central nervous system-assocaited bacterial pathogens in Nigeria
Source: Front Microbiol. 2026 Jun 16;17:1695489. doi: 10.3389/fmicb.2026.1695489 (PMC13314636; doi:10.3389/fmicb.2026.1695489)
Supplement: Supplementary file 2 [file Data_Sheet_2.DOCX]

**Supplementary Fig. 1. Supporting diagnostic, temporal, and species-resolution analyses across laboratories and specimen types in Nigeria.** (a) Diagnostic cascade per laboratory showing total cultures submitted, cultures processed/valid, and culture-positive isolates; the red line indicates laboratory-specific antimicrobial susceptibility testing (AST) coverage, highlighting inter-laboratory variability in diagnostic throughput and downstream resistance reporting. (b) Year-stratified distribution of the top ten specimen types, illustrating increasing urine and blood submissions over time and persistent underrepresentation of CSF. (c) Genus-level composition across all isolates, showing dominance of Staphylococcus species and greater species diversity within Klebsiella and Escherichia. (d) Temporal trends of the top 10 species, highlighting peaks in S. aureus and Klebsiella pneumoniae in 2017. (e) Species ambiguity index by genus showing high rates of non-speciated “sp.” entries among Neisseria, Cryptococcus, Listeria, and Acinetobacter, emphasizing limitations in species-level identification for key CNS pathogens.
